# Thalidomide increases both intra-tumoural tumour necrosis factor-α production and anti-tumour activity in response to 5,6-dimethylxanthenone-4-acetic acid

**DOI:** 10.1038/sj.bjc.6690415

**Published:** 1999-05

**Authors:** Z Cao, W R Joseph, W L Browne, K G Mountjoy, B D Palmer, B C Baguley, L-M Ching

**Affiliations:** Auckland Cancer Society Research Centre, and Research Centre for Developmental Medicine and Biology, University of Auckland School of Medicine, Auckland, New Zealand

**Keywords:** DMXAA, thalidomide, Colon 38, endotoxin, tumour necrosis factor

## Abstract

5,6-Dimethylxanthenone-4-acetic acid (DMXAA), synthesized in this laboratory and currently in phase I clinical trial, is a low molecular weight inducer of tumour necrosis factor-α (TNF-α). Administration of DMXAA to mice with established transplantable tumours elicits rapid vascular collapse selectively in the tumour, followed by extensive haemorrhagic necrosis mediated primarily through the production of TNF-α. In this report we have investigated the synthesis of TNF-α mRNA in hepatic, splenic and tumour tissue. Co-administration of thalidomide with DMXAA increased anti-tumour activity and increased intra-tumoural TNF-α production approximately tenfold over that obtained with DMXAA alone. Thalidomide increased splenic TNF-α production slightly but significantly decreased serum and hepatic levels of TNF-α induced with DMXAA. Lipopolysaccharide (LPS) induced 300-fold higher serum TNF-α than did DMXAA at the maximum tolerated dose, but induced similar amounts of TNF-α in spleen, liver and tumour. Splenic TNF-α activity induced with LPS was slightly increased with thalidomide, but serum and liver TNF-α levels were suppressed. Thalidomide did not increase intra-tumoural TNF-α production induced with LPS, in sharp contrast to that obtained with DMXAA. While thalidomide improved the anti-tumour response to DMXAA, it had no effect on the anti-tumour action of LPS that did not induce a significant growth delay or cures against the Colon 38 tumour. The increase in the anti-tumour action by thalidomide in combination with DMXAA corresponded to an increase in intra-tumoural TNF-α production. Co-administration of thalidomide may represent a novel approach to improving selective intra-tumoural TNF-α production and anti-tumour efficacy of DMXAA. © 1999 Cancer Research Campaign


*British Journal of Cancer* (1999) **80**(5/6), 716–723
© 1999 Cancer Research Campaign
Article no. bjoc.1998.0415

### Thalidomide increases both intra-tumoural tumour necrosis factor-α production and anti-tumour activity in response to 5,6-dimethylxanthenone-4-acetic acid

**Z Cao[1], WR Joseph[1], WL Browne[1], KG Mountjoy[2], BD Palmer[1], BC Baguley[1] and L-M Ching[1]**

[1]Auckland Cancer Society Research Centre and [2]Research Centre for Developmental Medicine and Biology, University of Auckland School of Medicine, Auckland, New Zealand

**Summary** 5,6-Dimethylxanthenone-4-acetic acid (DMXAA), synthesized in this laboratory and currently in phase I clinical trial, is a low molecular weight inducer of tumour necrosis factor-α (TNF-α). Administration of DMXAA to mice with established transplantable tumours elicits rapid vascular collapse selectively in the tumour, followed by extensive haemorrhagic necrosis mediated primarily through the production of TNF-α. In this report we have investigated the synthesis of TNF-α mRNA in hepatic, splenic and tumour tissue. Co-administration of thalidomide with DMXAA increased anti-tumour activity and increased intra-tumoural TNF-α production approximately tenfold over that obtained with DMXAA alone. Thalidomide increased splenic TNF-α production slightly but significantly decreased serum and hepatic levels of TNF-α induced with DMXAA. Lipopolysaccharide (LPS) induced 300-fold higher serum TNF-α than did DMXAA at the maximum tolerated dose, but induced similar amounts of TNF-α in spleen, liver and tumour. Splenic TNF-α activity induced with LPS was slightly increased with thalidomide, but serum and liver TNF-α levels were suppressed. Thalidomide did not increase intra-tumoural TNF-α production induced with LPS, in sharp contrast to that obtained with DMXAA. While thalidomide improved the anti-tumour response to DMXAA, it had no effect on the anti-tumour action of LPS that did not induce a significant growth delay or cures against the Colon 38 tumour. The increase in the anti-tumour action by thalidomide in combination with DMXAA corresponded to an increase in intra-tumoural TNF-α production. Co-administration of thalidomide may represent a novel approach to improving selective intra-tumoural TNF-α production and anti-tumour efficacy of DMXAA.

**Keywords:** DMXAA; thalidomide; Colon 38; endotoxin; tumour necrosis factor

Therapies that can selectively target the tumour vasculature represent an attractive approach to limiting the growth of tumours. 5,6-Dimethylxanthenone-4-acetic acid (DMXAA, Figure 1), synthesized in this laboratory, demonstrates excellent activity against murine tumours, inducing irreversible cessation of tumour blood flow within 4 h of administration with little effect on perfusion of normal tissues (Zwi et al, 1994; Lash et al, 1998). The ischaemia and haemorrhagic necrosis that follow provide a histological appearance similar to that of tumours treated with tumour necrosis factor-α (TNF-α) (Zwi et al, 1994). DMXAA elevates serum TNF-α activity in a manner that correlates with the anti-tumour response (Philpott et al, 1995), suggesting that the induction of TNF-α plays an essential role in its anti-tumour action. The anti-tumour activity and tumour blood flow effects of flavone acetic acid (FAA), a compound closely related to DMXAA, is inhibitable by antibodies to TNF-α (Mahadevan et al, 1990; Pratesi et al, 1990), further supporting this hypothesis.

In murine systems, FAA and DMXAA exhibit similar profiles of immune modulation, cytokine induction and anti-tumour activities, with the exception that DMXAA is 12-fold more potent than FAA (Ching et al, 1991; Rewcastle et al, 1991). However, only DMXAA up-regulates TNF-α mRNA in human cell lines (Ching et al, 1994*a*; Patel et al, 1997), and induces TNF-α production in cultured human peripheral blood cells (Philpott et al, 1997). Thus, DMXAA appears to be a superior clinical candidate to FAA in that it is more dose-potent and does not exhibit the mouse-human species preference of FAA. DMXAA is currently undergoing phase I clinical trial as a single agent.

Thalidomide, a small molecule with diverse pharmacological and biological effects, selectively inhibits TNF-α synthesis induced by lipopolysaccharide (LPS) (Moreira et al, 1993) and DMXAA (Ching et al, 1995). Thalidomide has attracted much interest in the treatment of leprosy (Sheskin, 1965; Sampaio et al, 1991), Behçet's disease (Gardner-Medwin et al, 1994) and pruritus

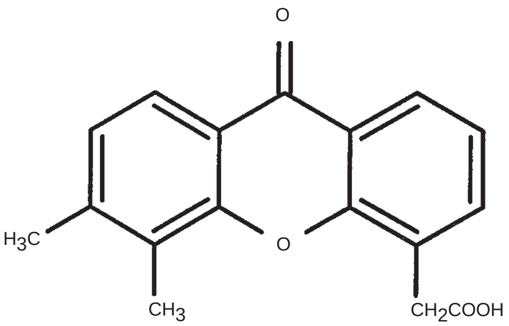

**Figure 1** Chemical structure of DMXAA

*Received 30 July 1998*
*Accepted 4 November 1998*

Correspondence to: L-M Ching, Auckland Cancer Society Research Centre, University of Auckland School of Medicine, Private Bag 92019, Auckland, New Zealand

(Silva et al, 1994), but was withdrawn from use as a sedative and anti-emetic in the 1960s because of its teratogenicity (Fabro et al, 1967). Recent studies indicate that its immunosuppressive and anti-inflammatory effects would be beneficial to the treatment of graft-versus-host disease (Vogelsang et al, 1992; Uthoff et al, 1995), rheumatoid arthritis (Gutierrez-Rodriguez et al, 1989), prevention of graft rejection (Vogelsang et al, 1988; Uthoff et al, 1995), HIV/AIDS (Makonkawkeyoon et al, 1993; Moreira et al, 1997), sarcoidosis (Carlesimo et al, 1995) and various other inflammatory diseases (Burroughs et al, 1995).

In experiments to determine whether suppression of serum TNF-α production by thalidomide affected the antitumour response to DMXAA, we found that, while serum TNF-α levels decreased, anti-tumour activity was unexpectedly improved (Ching et al, 1995). Reduction of tumour blood flow induced by DMXAA, which is thought to be mediated by TNF-α, was not reversed by thalidomide (Browne et al, 1998). Thalidomide analogues that were more potent than thalidomide in inhibiting DMXAA-induced serum TNF-α were also more potent in improving the anti-tumour response, indicating that the two activities were associated (Ching et al, 1998). Pentoxifylline, another inhibitor of TNF-α synthesis differing from thalidomide in both structure and mechanism of inhibition (Han et al, 1990; Moreira et al, 1993), also increased the anti-tumour action of DMXAA (Ching et al, 1998).

Results with LPS also raise questions about the relationship between TNF-α production and anti-tumour effect. While LPS is more effective than DMXAA as an inducer of serum TNF-α, and both induce haemorrhagic necrosis of Colon 38 tumours, only DMXAA induces long tumour growth delays and cures (Ching et al, 1994*b*). In this report, we consider whether TNF-α production in tissues, rather than in serum, provides an explanation of anti-tumour effects. We have combined the results of several techniques to investigate the effects of DMXAA and LPS in combination with thalidomide.

### MATERIALS AND METHODS

### Mice

C57B1/6 mice were purchased from the Department of Laboratory Animal Science, Otago Medical School, Dunedin, New Zealand. (C57B1/6 × DBA/2)$F_1$ hybrid mice were bred in our animal facility and were housed under conditions of constant temperature and humidity according to institutional ethical guidelines. All mice were used between 8 and 12 weeks of age.

### Materials

DMXAA, synthesized in this laboratory (Rewcastle et al, 1991), was dissolved in 5% sodium bicarbonate and injected in a volume of 0.01 ml $g^{-1}$ body weight. (C57B1/6 × DBA/2)$F_1$ mice and C57B1/6 mice were treated with DMXAA at their respective optimal therapeutic dose of 30 mg $kg^{-1}$ and 22.5 mg $kg^{-1}$. LPS (*Escherichia coli* serotype 055:B5, purchased from Sigma) was dissolved in water and injected at its maximum tolerated dose of 175 μg per 0.2 ml per mouse (Ching et al, 1995). (−)-Thalidomide was synthesized in this laboratory according to published methods (Casini and Ferappi, 1964), dissolved in dimethyl sulphoxide and injected at 2.5 μl $g^{-1}$ at a dose of 100 mg $kg^{-1}$.

### Tumour growth inhibition assays

(C57B1/6 × DBA/2)$F_1$ mice were implanted under anaesthesia (sodium pentobarbital, 90 mg $kg^{-1}$) subcutaneously in the left flank with Colon 38 tumour fragments. When the tumours were approximately 4 mm in diameter (generally 7–9 days after implantation), mice were treated intraperitoneally with drug. Tumours were measured thrice weekly after treatment and tumour volumes were calculated as $0.52\ a^2 \times b$, where a and b are the minor and major axes of the tumour. The arithmetic means and standard errors were calculated for each point, including animals having zero measured tumour volume, and expressed as a fraction of the pretreatment volume. Growth delay was determined as the difference in the number of days required for the untreated, control and treated tumours to reach four times the pretreatment volume. Six mice were used for each group, and mice in which the tumour had completely disappeared were kept for at least 3 months to ensure that the tumours did not re-grow.

### In situ hybridization

C57B1/6 mice with tumours approximately 6 mm in diameter were treated with DMXAA (22.5 mg $kg^{-1}$), or LPS (175 μg per mouse) and, after 4 h, asphyxiated with nitrogen, and tissues fixed by whole body perfusion of paraformaldehyde in borate buffer. Tissues were further fixed after removal, snap-frozen in isopentane in liquid nitrogen, and cryosections (15 μM) were cut. The mounted sections were further fixed with paraformaldehyde, deproteinized using proteinase K, equilibrated in triethanolamine to block positive charges, and dehydrated with ascending concentrations of ethanol. $^{33}P$-labelled antisense riboprobe ($2 \times 10^7$ cpm $ml^{-1}$) in hybridization buffer (50% formamide, 0.2 M sodium chloride, 1 × Denhardt's solution, 1 mM EDTA, 2 mM Tris–HCl, 0.1% dextran sulphate, 0.1 mg $ml^{-1}$ tRNA, 2 mM dithiothreitol) was overlaid on the sections, cover-slipped and sealed, and hybridized overnight at 65°C. After hybridization, the sections were digested with RNAase (10 mg $ml^{-1}$, 30 min at 37°C), washed twice (5 min) with 2 × standard saline citrate (SSC), once (10 min) with 1 × SSC, and once (10 min) with 0.5 × SSC, all at room temperature. Final high stringency washes were carried out using 0.1 × SSC for 30 min at 65°C, followed by a final wash with 0.1 × SSC (5 min at room temperature). Sections were dehydrated through ethanol plus 1 × SSC, air-dried and processed for autoradiography. After 2–3 weeks the slides were developed and stained with haematoxylin and eosin. Sections of tissues from untreated mice were processed in an identical manner. The antisense murine TNF-α riboprobe was transcribed from a cDNA template encoding 700 base pairs of the murine TNF-α gene. Probes were radiolabelled using $^{33}P$-UTP and a Riboprobe Gemini II labelling kit from Promega.

### Northern analysis

Organs were excised from sacrificed mice, minced using scalpel blades, and total cellular RNA was extracted using Trizol (Gibco BRL) according to manufacturer's instructions. The RNA samples (10 μg) were fractionated by electrophoresis on a formaldehyde-denaturing 1% agarose gel and transferred overnight to a nylon membrane (Hybond $N^+$, Amersham). After UV cross-linking (120 mJoule, UV-Stratalinker, Stratagene, San Diego, CA, USA), the membrane was baked (30 min, 78°C), and each membrane was

© Cancer Research Campaign 1999

pre-hybridized (2 h, 42°C) in 7 ml hybridization mix (50% formamide, 0.075 M sodium chloride, 0.05 M sodium dihydrogen phosphate, 5 mM EDTA, 0.001% polyvinyl pyrrolidone, 0.001% bovine serum albumin, 0.001% Ficoll, 0.01 mg $ml^{-1}$ herring sperm DNA and 0.5% sodium dodecyl sulphate (SDS)). The cDNA to murine TNF-α mRNA was labelled with $\alpha^{32}$P-dCTP (Amersham) using a random priming kit (RTS Radprime DNA labelling system, Gibco BRL). Excess radioactivity was removed by elution through a G-50 Sephadex column and labelled probe ($10^6$ cpm $ml^{-1}$ hybridization mix) was then added to the membrane and hybridized for 36 h at 42°C. The blots were washed twice in 2 × SSC with 0.1% SDS for 10 min at 42°C, and finally in 0.2 × SSC with 0.1% SDS for 10 min at 65°C. Blots were exposed to X-ray film for 1–3 days at −70°C. Membranes were then stripped (two washes in 300 ml 0.1 × SSC with 1% SDS for 15 min at 80°C) and re-hybridized with probe for human β-actin to determine loading of the lanes. Intensity of signals was quantitated by laser densitometric scanning.

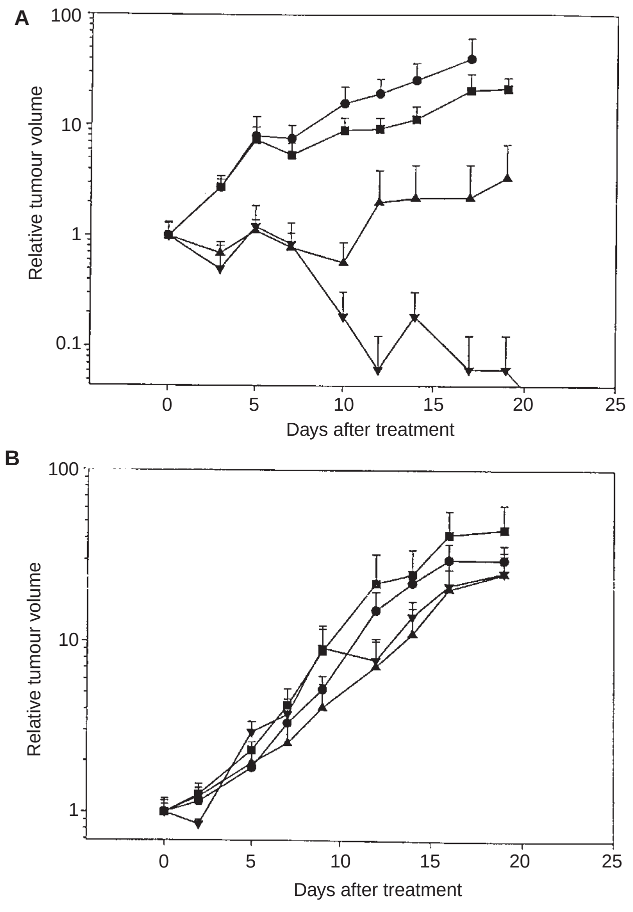

**Figure 2** Effect of thalidomide on the anti-tumour action of DMXAA (**A**) and LPS (**B**). Results for DMXAA are redrawn from a previously published study (Ching et al, 1995) to allow comparison. (C57B1/6 × DBA/2)$F_1$ mice with Colon 38 tumours were treated with drugs and the growth of tumours followed. Untreated tumours, (●); thalidomide (100 mg $kg^{-1}$ alone (■); DMXAA (30 mg $kg^{-1}$ or LPS (175 μg per mouse) alone (▲) or in combination with thalidomide (▼). Six mice per group

### TNF-α determination

Blood was collected from the ocular sinus from mice anaesthetized with halothane, coagulated overnight at 4°C then centrifuged for 30 min at 2000 ***g*** at 4°C. Extractable TNF-α in spleen, liver and tumour was measured using a previously published method (Yang et al, 1994). Tissues were excised, weighed and homogenized in α-modified minimal essential medium (2 ml) using a tissue homogenizer. Homogenates were centrifuged at 2000 ***g*** for 30 min at 4°C, and the supernatant was removed and centrifuged at 14 000 ***g*** for 30 min at 4°C before assay. TNF-α activity was measured using the standard L929 cytotoxicity assay (Hogan and Vogel, 1990; Philpott et al, 1995). L929 cells (3 × $10^4$ per well) were allowed to adhere overnight to the bottom of flat-bottomed 96-well plates. The cells were then sensitized with Actinomycin D (8 μg $ml^{-1}$ final concentration) for 1 h before the addition of serial dilutions of the samples to be assayed. Cell killing was assessed after 24 h by a colourimetric assay using 3-(4,5-dimethyl-2-thiazolyl)-2,5-diphenyl-2H-tetrazolium bromide as previously described (Philpott et al, 1995). One unit of TNF-α was defined as that required to produce 50% killing of L929 cells.

### RESULTS

### Comparative effects of thalidomide on the anti-tumour action of DMXAA and LPS

We first investigated whether thalidomide could improve the anti-tumour action of LPS, another inducer of TNF-α, as it does with DMXAA (Ching et al, 1995). As shown in Figure 2, thalidomide did not improve the response to LPS; rather the tumours in the groups receiving thalidomide only or in combination with LPS grew at a slightly faster rate than the untreated tumours. LPS induced a growth delay of 3 days, and one of the mice in the group of six was cured. By comparison, DMXAA alone induced a growth delay of 17 days and a cure rate of 83%, and in combination with thalidomide, all the mice were cured.

### Effect of thalidomide on TNF-α mRNA and protein production induced by DMXAA or LPS

C57B1/6 mice with Colon 38 tumours were treated with DMXAA (22.5 mg $kg^{-1}$) or LPS (175 μg per mouse), with or without thalidomide (100 mg $kg^{-1}$). Mice were bled, and spleen, liver and tumours excised 1–5 h after treatment. Serum was prepared from the blood samples for TNF-α measurements and organs were analysed for both TNF-α mRNA using Northern blots, and for TNF-α activity using a bioassay. DMXAA up-regulated TNF-α mRNA in spleen, liver and tumour within 1 h, with maximal activity at 2 h and declining thereafter. Co-administration of thalidomide resulted in a sustained and heightened transcription of TNF-α mRNA in the spleen and tumour, with TNF-α mRNA elevated to 5 h after treatment (Figure 3A). In the liver, thalidomide suppressed transcription as well as delaying the peak to 4 h (Figure 3A). LPS also up-regulated TNF-α mRNA in spleen, liver and tumour, and thalidomide suppressed transcription in all three tissues with no apparent alteration of the kinetics (Figure 3B). Thalidomide alone did not induce TNF-α mRNA in any of the tissues at 2 h (Figure 3 A, B).

DMXAA and LPS induced TNF-α protein, as measured by bioassay, in the serum, spleen, liver and tumour (Figure 4). LPS

 © Cancer Research Campaign 1999

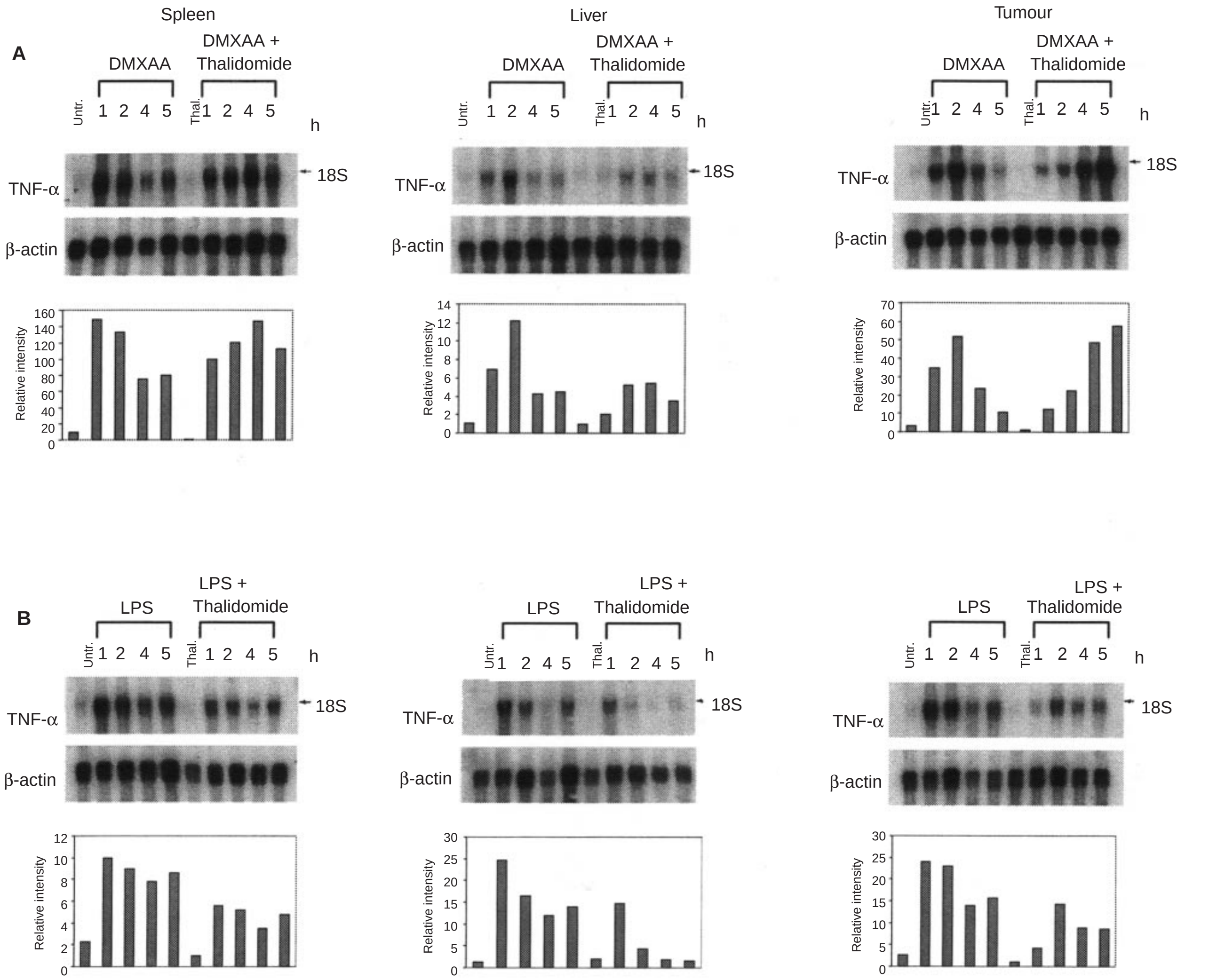

**Figure 3** Effect of thalidomide on TNF-α mRNA up-regulation induced with DMXAA (**A**) and LPS (**B**). C57B1/6 mice with Colon 38 tumours were treated with DMXAA (22.5 mg $kg^{-1}$) or LPS (175 μg per mouse) alone or in combination with thalidomide (100 mg $kg^{-1}$). Tissues were excised after the times indicated and processed for Northern blot analysis of TNF-α mRNA expression. Negative controls were from untreated mice (Untr) or from mice 2 h after receiving thalidomide only (Thal). Tissues were pooled from three mice per group. The intensity of signals was quantitated, normalized by comparison with the signal for human β-actin to determine loading of the lanes, and expressed as a bar graph induced 300-fold higher amounts of serum TNF-α than DMXAA with a peak 1 h after administration. Tissue TNF-α activity was high 1 h after LPS administration and it is possible that some of the measured TNF-α activity derived from contaminating blood since tissues were not perfused before extraction. The differential effects of thalidomide on TNF-α activity were in general more pronounced than those observed for TNF-α mRNA (Figure 4). In serum, spleen and liver, thalidomide delayed peak TNF-α activity following DMXAA from 2 h to 4 h and suppressed peak activity in serum and liver. TNF-α activity in the tumour increased with time after DMXAA treatment and peak activity was dramatically increased (ninefold) by thalidomide co-administration (Figure 4). In the case of LPS, thalidomide suppressed TNF-α responses in serum, spleen and tumour, and delayed the peak activity by 1–2 h. Thalidomide slightly increased TNF-α activity in the spleen.

### Effect of thalidomide on the number of cells in the tumour expressing TNF-α mRNA after DMXAA

The increased TNF-α mRNA in tumour tissue caused by co-administration of thalidomide with DMXAA (Figure 4) could result from either increased transcription or an increased proportion of responding cells. Sections of tumour were therefore taken 4 h after treatment from mice treated with DMXAA (22.5 mg $kg^{-1}$) with or without thalidomide (100 mg $kg^{-1}$), and cells expressing TNF-α mRNA were identified by in situ hybridization to an antisense riboprobe for TNF-α. Tumour tissue from control animals and from animals treated with thalidomide alone showed no labelling of cells. Tumour tissue from mice treated with DMXAA with or without thalidomide were both heavily labelled, but the number of labelled cells was similar (Figure 5).

© Cancer Research Campaign 1999

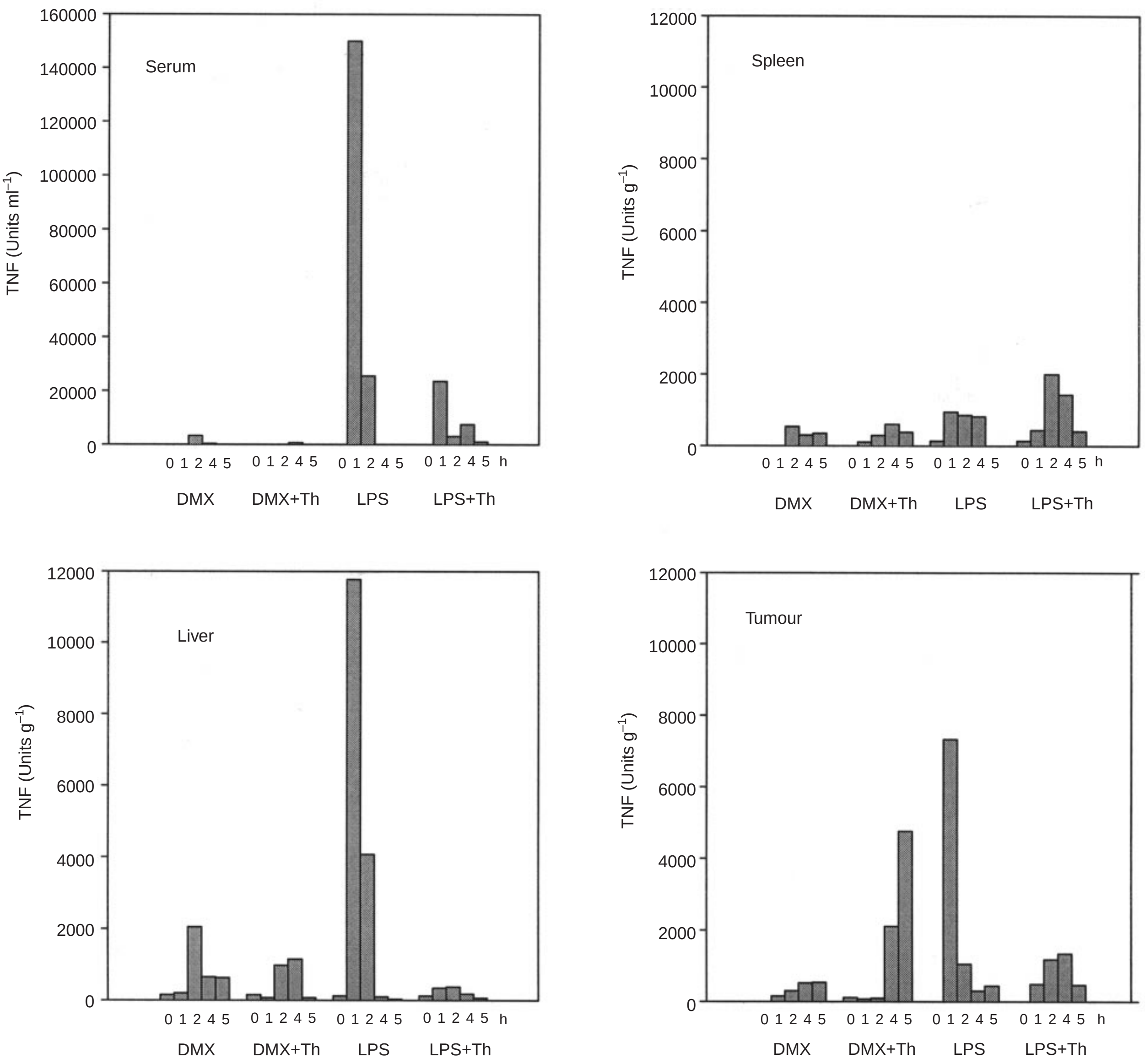

**Figure 4** Effect of thalidomide on TNF-α activity in serum, spleen, liver and tumour taken at the indicated times from mice treated with DMXAA (DMX), DMXAA plus thalidomide (DMX + Th), LPS and LPS plus thalidomide (LPS + Th). A sample of same tissue used for the Northern analyses in Figure 3 was homogenized in 2 ml culture medium and the supernatants assayed for TNF-α activity. Serum obtained from the same pool of mice was also assayed for TNF-α activity. Activity was normalized to units TNF-α per gram of tissue homogenized or per ml of serum

### DISCUSSION

We have investigated differences in TNF-α production in the tumour and systemic organs induced with LPS or DMXAA, with or without thalidomide, to obtain an explanation for why thalidomide, which suppresses the serum TNF-α response to both LPS and DMXAA, potentiates the anti-tumour activity only of DMXAA. Thalidomide increases the peak intra-tumoural TNF-α levels induced with DMXAA approximately tenfold while decreasing LPS-induced tumour TNF-α by sixfold. It also suppresses liver and serum TNF-α activity induced by LPS and DMXAA, while slightly elevating splenic TNF-α production. Thalidomide also delays the kinetics of TNF-α production from a peak in the normal tissues at 2 h to 4 h after DMXAA treatment, and from 1 h to 2 h after LPS (Figure 4).

The TNF-α activity in tumour tissue induced after 1 h by LPS is much higher than that induced by DMXAA (Figure 4), demonstrating that tumour-associated TNF-α per se does not correlate with anti-tumour effect. Early (1–2 h) TNF-α synthesis may be related to the induction of tumour haemorrhagic necrosis, which is common to the action of both LPS and DMXAA (Ching et al, 1994*b*). On the other hand, the destruction of all tumour cells required for tumour regression may require the presence of tumour-associated TNF-α over a longer time period, as seen with DMXAA. Examination of tumours treated with a sub-therapeutic dose of DMXAA shows that co-administration of thalidomide decreases surviving tumour cells (Ching et al, 1998), consistent with this hypothesis. The results of Northern analysis (Figure 2), combined with the in situ hybridization data (Figure 5), show that co-administration of thalidomide with DMXAA does not increase

 *© Cancer Research Campaign 1999*

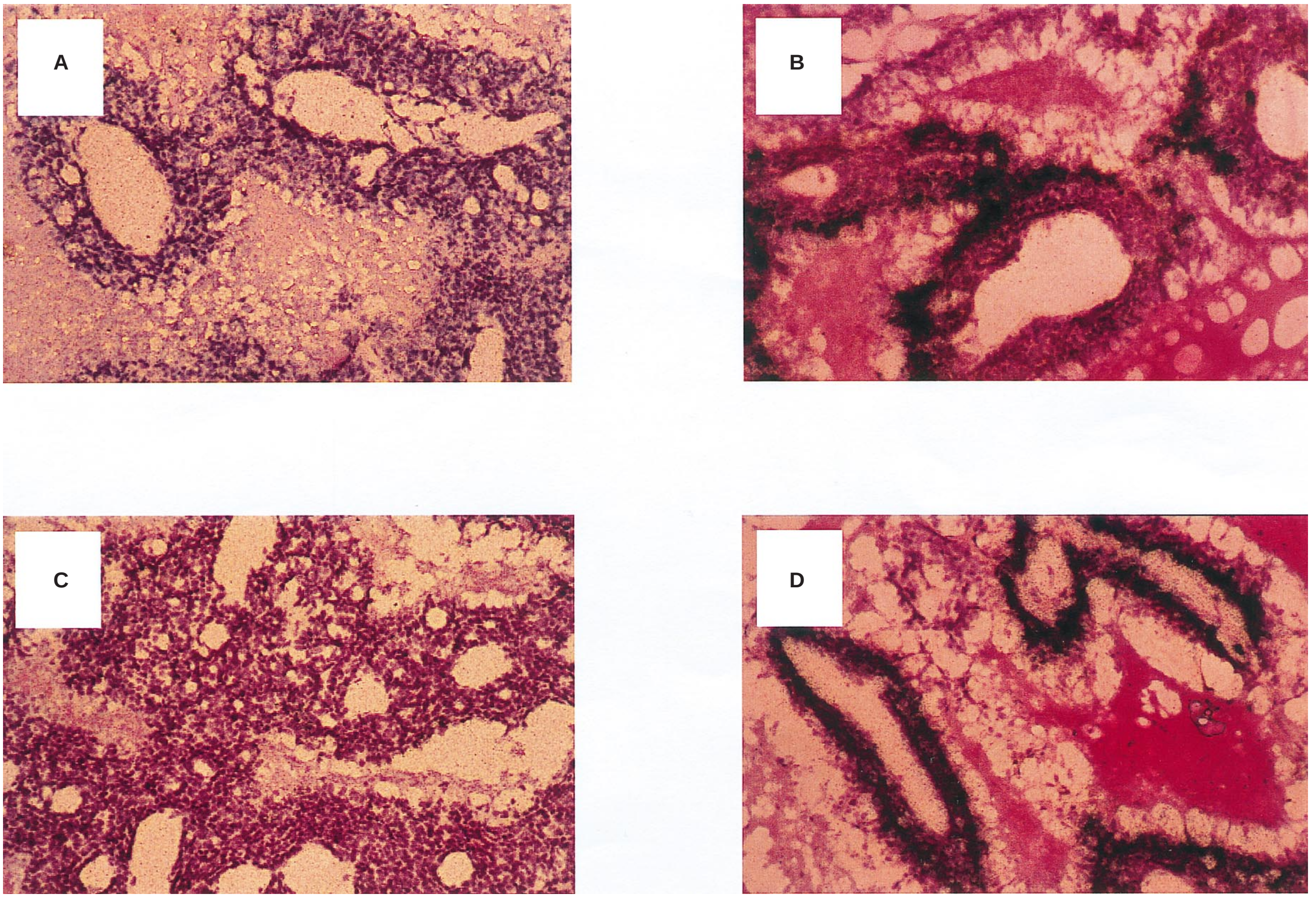

**Figure 5** In situ hybridization, using an antisense probe for TNF-α, of Colon 38 tumour sections taken from C57B1/6 mice that were either untreated (**A**), treated 4 h previously with DMXAA alone (22.5 mg $kg^{-1}$) (**B**), treated 4 h previously with thalidomide alone (100 mg $kg^{-1}$) (**C**), or treated 4 h previously with DMXAA in combination with thalidomide (**D**)

the number of cells expressing TNF-α mRNA. Rather, elevation of intra-tumoural TNF-α activity appears to be post-transcriptional, accentuated by TNF-α entrapment following vascular collapse.

The reason for the dramatic contrast in the effect of thalidomide on intra-tumoural TNF-α activity induced with LPS or DMXAA is not clear. In the tumour, DMXAA and LPS may activate different cell populations. We have obtained evidence that DMXAA activates a broader range of cells than LPS and induces the tumour cells themselves to produce TNF-α (Joseph et al, 1999). The activity of LPS would be expected to be limited to cells such as the host macrophages that express surface receptors that bind to it. Moreover, different cell types may be differently regulated by thalidomide. Thalidomide is noted for its inhibition of TNF-α production by LPS-activated human blood monocytes in culture (Sampaio et al, 1991), but bi-directional dose-dependent TNF-α modulation has been reported, depending on the cell type and stimulator used. Thalidomide enhanced TNF-α synthesis by the HL-60 human leukaemia cell line induced with phorbol esters, but inhibited TNF-α synthesis induced with okadaic acid in the same cell line (Miyachi et al, 1996). While thalidomide inhibited synthesis of TNF-α in LPS-stimulated cultures of unfractionated human blood mononuclear cells, it enhanced TNF-α synthesis in cultures of THP-1 cells and in the adherent population of human blood mononuclear cells (Shannon and Sandoval, 1996).

The mechanism by which thalidomide modulates TNF-α production is still unclear. Thalidomide selectively enhanced the degradation of TNF-α mRNA (Moreira et al, 1993), but later work suggests that thalidomide inhibits HIV replication in macrophages through decreased binding activity of NFκB (Moreira et al, 1997), an important transcription factor controlling activation of the TNF-α gene (Shakov et al, 1990). As shown by photoaffinity-labelled thalidomide, thalidomide binds to $\alpha_1$-acid glycoproteins that have intrinsic anti-TNF-α activity (Turk et al, 1996).

In addition to regulating TNF-α synthesis, thalidomide has a number of other effects that could contribute to the anti-tumour response, including modulation of T lymphocytes, alteration of pharmacokinetics and metabolism, and inhibition of angiogenesis. The anti-tumour action of DMXAA against the Colon 38 tumour is more efficient in immunocompetent hosts, indicating that host T-cell immunity is beneficial to tumour regression (Ching et al, 1992). Although alterations to T-cell subsets by thalidomide were not observed in one study (Shannon et al, 1994), co-stimulation of purified primary human T lymphocytes was shown in another (Haslett et al, 1998). We are actively investigating the effects of thalidomide on the pharmacokinetics of DMXAA. Thalidomide inhibits angiogenesis (D'Amato et al, 1994), and combination of angiogenesis antagonists with conventional anticancer agents can improve anti-tumour activity (Teicher et al, 1992). However, inhibition of angiogenesis by thalidomide generally requires repeated

© Cancer Research Campaign 1999 

administration and we have not observed any action of thalidomide, given either as single or multiple injections, on the growth of Colon 38 tumours (Ching et al, 1998).

In conclusion, our results, together with those from studies on thalidomide analogues (Ching et al, 1998), strongly suggest that modulation of the magnitude and kinetics of intra-tumoural TNF-α production by thalidomide is the basis for its enhancement of the anti-tumour action of DMXAA. Thalidomide, because of its ability to increase anti-tumour efficacy whilst reducing serum TNF-α activity, might be considered as an adjunct to DMXAA in future clinical trials.

### ACKNOWLEDGEMENTS

The authors gratefully acknowledge the assistance of Steven Edgar, Department of Pathology, University of Auckland in the preparation of the photographs.

### REFERENCES

Browne WL, Wilson WR, Rutland M, Baguley BC and Ching L-M (1998) Suppression of serum tumour necrosis factor-α by thalidomide does not lead to reversal of tumour vascular collapse and anti-tumour activity of 5,6-dimethylxanthenone-4-acetic acid. *Anticancer Res* **18**: 4409–4414

Burroughs MH, Tsenova-Berkova L, Sokol K, Ossig J, Tuomanen E and Kaplan G (1995) Effect of thalidomide on the inflammatory response in cerebrospinal fluid in experimental bacterial meningitis. *Microb Pathogen* **19**: 245–255

Carlesimo M, Giustini S, Rossi A, Bonaccorsi P and Calvieri S (1995) Treatment of cutaneous and pulmonary sarcoidosis with thalidomide. *J Am Acad Dermatol* **32**: 866–869

Casini G and Ferappi M (1964) Preparation of one optical antipode of 2-phthalimidoglutarimide. *Farmaco* **19**: 563–565

Ching LM, Joseph WR, Zhuang L, Atwell GJ, Rewcastle GR, Denny WA and Baguley BC (1991) Induction of natural killer activity by xanthenone analogues of flavone acetic acid: relation with anti-tumour activity. *Eur J Cancer* **27**: 79–83

Ching LM, Joseph WR and Baguley BC (1992) Anti-tumour responses to flavone-8-acetic acid and 5,6-dimethylxanthenone-4-acetic acid in immune deficient mice. *Br J Cancer* **66**: 128–130

Ching LM, Joseph WR, Crosier KE and Baguley BC (1994*a*) Induction of tumour necrosis factor-alpha messenger RNA in human and murine cells by the flavone acetic acid analogue 5,6-dimethylxanthenone-4-acetic acid (NSC 640488). *Cancer Res* **54**: 870–872

Ching LM, Joseph WR, Zhuang L and Baguley BC (1994*b*) Interaction between endotoxin and the antitumour agent 5,6-dimethylxanthenone-4-acetic acid in the induction of tumour necrosis factor and haemorrhagic necrosis of Colon 38 tumours. *Cancer Chemother Pharmacol* **35**: 153–160

Ching LM, Xu Z-F, Gummer BH, Palmer BD, Joseph WR and Baguley BC (1995) Effect of thalidomide on tumour necrosis factor production and anti-tumour activity induced by 5,6-dimethylxanthenone-4-acetic acid. *Br J Cancer* **72**: 339–343

Ching LM, Browne WL, Tchernegovski R, Gregory T, Baguley BC and Palmer BD (1998) Interaction of thalidomide, phthalimide analogues of thalidomide and pentoxifylline with the antitumour agent 5,6-dimethylxanthenone-4-acetic acid: concomitant reduction of serum tumour necrosis factor-alpha and enhancement of antitumour activity. *Br J Cancer* **78**: 336–343

D'Amato RJ, Loughnan MS, Flynn E and Folkman J (1994) Thalidomide is an inhibitor of angiogenesis. *Proc Natl Acad Sci USA* **91**: 4082–4085

Fabro S, Smith RL and Williams RT (1967) Toxicity and teratogenicity of optical isomers of thalidomide. *Nature* **215**: 296

Gardner-Medwin JM, Smith NJ and Powell RJ (1994) Clinical experience with thalidomide in the management of severe oral and genital ulceration in conditions such as Behçet's disease: use of neurophysiological studies to detect thalidomide neuropathy. *Ann Rheum Dis* **53**: 828–832

Gutierrez-Rodriguez O, Starusta-Bacal P and Gutierrez-Montes O (1989) Treatment of refractory rheumatoid arthritis: the thalidomide experience. *J Rheumatol* **16**: 158–163

Han J, Thompson P and Beutler B (1990) Dexamethasone and pentoxifylline inhibit endotoxin-induced cachectin/tumor necrosis factor synthesis at separate points in the signaling pathway. *J Exp Med* **172**: 391–394

Haslett PAJ, Corral LG, Albert M and Kaplan G (1998) Thalidomide costimulates primary human t lymphocytes, preferentially inducing proliferation, cytokine production, and cytotoxic responses in the cd8+ subset. *J Exp Med* **187**: 1885–1892

Hogan MM and Vogel SN (1990) Measurement of tumor necrosis factor alpha and beta. In *Current Protocols in Immunology*, Vol. 1, Coligan JE, Kruisbeek AM, Margulies DH, Shevach EM and Strober W (eds), p. 6.10.1. Greene Publishing Associates and Wiley Interscience: New York

Joseph WR, Cao Z, Mountjoy KG, Marshall ES, Baguley BC and Ching LM (1999) Stimulation of tumors to synthesize tumor necrosis factor-alpha *in situ* using 5,6-dimethylxanthenone-4-acetic acid: a novel approach to cancer therapy. *Cancer Res* **59**: 633–638

Lash CJ, Li AE, Rutland M, Baguley BC, Zwi LJ and Wilson WR (1998) Enhancement of the anti-tumour effects of the antivascular agent 5,6-dimethylxanethenone-4-acetic acid (DMXAA) by combination with 5-hydroxytryptamine and bioreductive drugs. *Br J Cancer* **78**: 439–445

Mahadevan V, Malik STA, Meager A, Fiers W, Lewis GP and Hart IR (1990) Role of tumor necrosis factor in flavone acetic acid-induced tumor vasculature shutdown. *Cancer Res* **50**: 5537–5542

Makonkawkeyoon S, Limson-Pobre RNR, Moreira AL, Schauf V and Kaplan G (1993) Thalidomide inhibits the replication of human immunodeficiency virus type 1. *Proc Natl Acad Sci USA* **90**: 5974–5978

Miyachi H, Azuma A, Hioki E, Iwasaki S, Kobayashi Y and Hashimoto Y (1996) Inducer-specific bidirectional regulation by thalidomide and phenylphthalimides of tumor necrosis factor-alpha production. *Biochem Biophys Res Commun* **224**: 426–430

Moreira AL, Sampaio EP, Zmuidzinas A, Frindt P, Smith KA and Kaplan G (1993) Thalidomide exerts its inhibitory action on tumour necrosis factor alpha by enhancing mRNA degradation. *J Exp Med* **177**: 1675–1680

Moreira AL, Corral LG, Ye W, Johnson B, Stirling D, Muller GW, Freedman VH and Kaplan G (1997) Thalidomide and thalidomide analogs reduce HIV type 1 replication in human macrophages in vitro. *AIDS Res Hum Retroviruses* **13**: 857–863

Patel S, Parkin SM and Bibby MC (1997) The effect of 5,6-dimethylxanthenone-4-acetic acid on tumor necrosis factor production by human immune cells. *Anticancer Res* **17**: 141–150

Philpott M, Baguley BC and Ching L-M (1995) Induction of tumour necrosis factor-alpha by single and repeated doses of the antitumour agent 5,6-dimethylxanthenone-4-acetic acid. *Cancer Chemother Pharmacol* **36**: 143–148

Philpott M, Joseph WR, Crosier KE, Baguley BC and Ching LM (1997) Production of tumour necrosis factor-alpha by cultured human peripheral blood leucocytes in response to the antitumour agent 5,6-dimethylxanthenone-4-acetic acid (NSC 640488). *Br J Cancer* **76**: 1586–1591

Pratesi G, Rodolfo M, Rovetta G and Parmiani G (1990) Role of T cells and tumour necrosis factor in antitumour activity and toxicity of flavone acetic acid. *Eur J Cancer* **26**: 1079–1083

Rewcastle GW, Atwell GJ, Zhuang L, Baguley BC and Denny WA (1991) Potential antitumor agents. 61. Structure–activity relationships for in vivo colon-38 activity among disubstituted 9-oxo-9H-xanthene-4-acetic acids. *J Med Chem* **34**: 217–222

Sampaio EP, Sarno EN, Galilly R, Cohn ZA and Kaplan G (1991) Thalidomide selectively inhibits tumor necrosis factor-alpha production by stimulated human monocytes. *J Exp Med* **173**: 699–703

Shakhov AN, Collart MA, Vassalli P, Nedospasov SA and Jongeneel CV (1990) Kappa B-type enhancers are involved in lipopolysaccharide-mediated transcriptional activation of the tumor necrosis factor alpha gene in primary macrophages. *J Exp Med* **171**: 35–47

Shannon EJ and Sandoval F (1996) Thalidomide can be either agonistic or antagonistic to LPS evoked synthesis of TNF-alpha by mononuclear cells. *Immunopharmacol Immunotoxicol* **18**: 59–72

Shannon EJ, Howe RC, McLean K and Hastings RC (1994) Thalidomide does not perturb CD2, CD4, CD5, CD8, HLA-DR, or HLA-A, B, C molecules in vitro on the membranes of cells with immune potential. *Immunopharmacol Immunotoxicol* **16**: 717–729

Sheskin J (1965) Further observation with thalidomide in lepra reactions. *Leprosy Rev* **36**: 183–187

Silva SRB, Viana PCF, Lugon NV, Hoette M, Ruzany F and Lugon JR (1994) Thalidomide for the treatment of uremic pruritus: a crossover randomised double-blind trial. *Life Sci* **31**: 270–273

 © Cancer Research Campaign 1999

Teicher BA, Sotomayor EA and Huang ZD (1992) Antiangiogenic agents potentiate cytotoxic cancer therapies against primary and metastatic disease. *Cancer Res* **52**: 6702–6704

Turk BE, Jiang H and Liu JO (1996) Binding of thalidomide to alpha(1)-acid glycoprotein may be involved in its inhibition of tumor necrosis factor alpha production. *Proc Natl Acad Sci USA* **93**: 7552–7556

Uthoff K, Zehr KJ, Gaudin PB, Kumar P, Cho PW, Vogelsang G, Hruban RH, Baumgartner WA and Stuart RS (1995) Thalidomide as replacement for steroids in immunosuppression after lung transplantation. *Ann Thorac Surg* **59**: 277–282

Vogelsang GB, Santos GW, Colvin OM and Chen T (1988) Thalidomide for graft-versus-host disease. *Lancet* **1**: 827

Vogelsang GB, Farmer ER, Hess AD, Altamonte V, Beschorner WE, Jabs DA, Corio RL, Levin LS, Colvin OM, Wingard JR and Santos GW (1992) Thalidomide for the treatment of chronic graft-versus-host disease. *New England J Med* **326**: 1055–1058

Yang D, Satoh M, Ueda H, Tsukagoshi S and Yamazaki M (1994) Activation of tumor-infiltrating macrophages by a synthetic lipid A analog (ONO-4007) and its implication in antitumor effects. *Cancer Immunol Immunother* **38**: 287–293

Zwi LJ, Baguley BC, Gavin JB and Wilson WR (1994) Correlation between immune and vascular activities of xanthenone acetic acid antitumor agents. *Oncol Res* **6**: 79–85

*© Cancer Research Campaign 1999* 
